# Correlation between paddy rice growth and satellite-observed methane column abundance does not imply causation

**DOI:** 10.1038/s41467-021-21434-7

**Published:** 2021-02-19

**Authors:** Zhao-Cheng Zeng, Brendan Byrne, Fang-Ying Gong, Zhonghua He, Liping Lei

**Affiliations:** 1 Joint Institute for Regional Earth System Science & Engineering, University of California, Los Angeles, Los Angeles, CA USA; 2 Division of Geological and Planetary Sciences, California Institute of Technology, Pasadena, CA USA; 3 Jet Propulsion Laboratory, California Institute of Technology, Pasadena, CA USA; 4 Zhejiang Climate Center, Hanzhou, China; 5 Key Laboratory of Digital Earth Science, Aerospace Information Research Institute, Chinese Academy of Sciences, Beijing, China

**Keywords:** Climate sciences, Environmental sciences

**Arising from** Zhang et al. *Nature Communications* (10.1038/s41467-019-14155-5) (2020)

In a recent study, Zhang et al.^1^ found paddy rice area and growth were strongly correlated with CH_4_ column-averaged dry-air mole fractions (XCH_4_) observed from satellites in Monsoon Asia. Based on these correlations, they argued that the spatial area and growth cycle of paddy rice drive the spatial distribution and seasonality of XCH_4_ in the region of the rice paddies. Here, by reanalyzing satellite XCH_4_ observations and running CH_4_ simulations with a chemical transport model, we show that (1) local variation in XCH_4_ is primarily driven by large scale CH_4_ flux signals advected into the local area rather than from local emission, indicating that variations in XCH_4_ do not simply translate to variations in the underlying rice paddy emissions. (2) Spatial correlations between rice paddy extent and XCH_4_ are confounded by cross-correlation with other XCH_4_ emission sources that have similar spatial structures. As a result, the spatial and temporal consistencies between rice paddies and XCH_4_ reported in Zhang et al.^1^ do not imply a causal relationship. The inference of emissions based on the correlation may lead to incorrect conclusions on the annual variabilities of rice paddy CH_4_ emissions in Monsoon Asia.

The space-based instruments Greenhouse gases Observing SATellite Thermal And Near-infrared Sensor for carbon Observation—Fourier Transform Spectrometer (GOSAT TANSO-FTS) and SCanning Imaging Absorption spectroMeter for Atmospheric CHartographY (SCIAMACHY) measure the column-averaged dry-air mole fraction of CH_4_, which is the ratio of vertical column densities (VCDs) between CH_4_ and dry air weighted by a column averaging kernel^2,3^. VCD is defined as the total number of molecules per unit area in a vertical column from the surface to the top of the atmosphere. The XCH_4_ observed from space is given by

1 $${\rm{XCH}}_{4} = f\left(\frac{{\rm{CH}}_{4}{\rm{VCD}}}{{\rm{DryAirVCD}}}\right)$$

in which $f( \cdot )$ is the satellite measurement operator for averaging kernel convolution. Therefore, the variability of XCH_4_ is subjected to any possible changes of CH_4_ at different altitudes due to atmospheric transport, apart from the surface layer emissions. Previous studies have shown the significant impact of long-range transport on XCO_2_ variability^4–6^) and the same mechanism can be applied to XCH_4_. In the northern extratropics, the atmospheric zonal mixing time is estimated to be about 2 weeks^7^, which is much shorter than the seasonal cycle of CH_4_ fluxes. As a result, the seasonal variability of XCH_4_ observed at any specific location can be driven by the large scale advected signal instead of the local signal from the underlying surface methane flux. To quantify the relative contribution of a local and external signal, we conducted a tagged tracer simulation using the greenhouse gas framework-Flux (GHGF-Flux) forward model (see “Methods“ and Supplementary Fig. 1) for the four regions of interest (ROIs) in Zhang et al.^1^, including Northeast China, Southeast China, North Bangladesh, and North India, as shown in Fig. 1a. We can see that the external contribution to the seasonal cycle of XCH_4_ outweighs the local contribution for Northeast China, Southeast China, and North India, while they are comparable in North Bangladesh. To further investigate the drivers of XCH_4_ seasonal variability, Fig. 1b shows the monthly averaged XCH_4_ in the four ROIs and the corresponding zonal means of XCH_4_ over latitudinal bands centered on the ROIs. The local XCH_4_ seasonal variabilities are strongly correlated (*p* value < 0.01) with the zonal mean seasonal cycle in all four ROIs despite considerable scatter due to retrieval error and synoptic-scale XCH_4_ variability. Such agreement is expected since the seasonal cycle of XCH_4_ has previously been shown to have strong zonal features (Supplementary Fig. 2; ref. ^3^). These two pieces of evidence strongly suggest that the local variability in XCH_4_ has a much larger footprint than the underlying local region. Therefore, any causal argument for a correlation between XCH_4_ observations from space and local surface emissions needs to account the effect of long-range atmospheric transport.

**Fig. 1:**
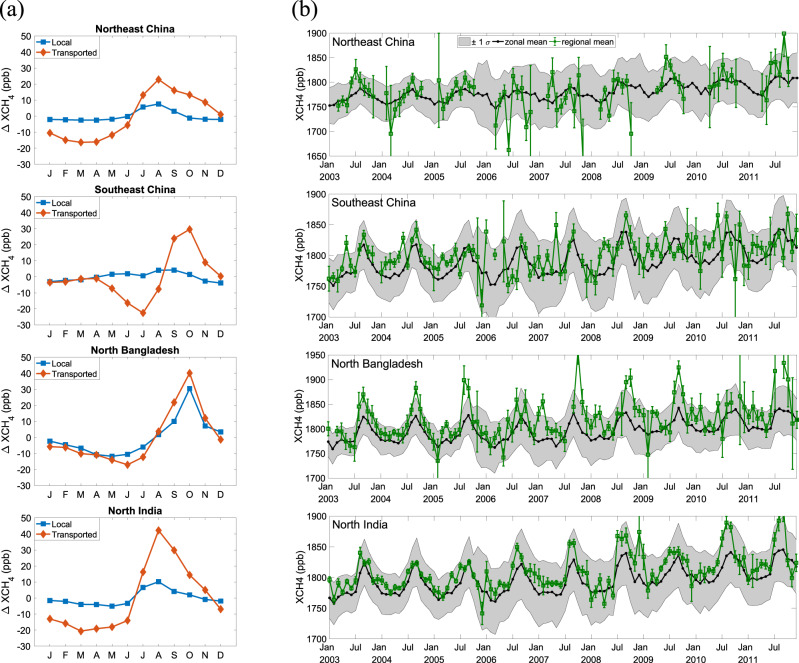
Measured and modeled monthly XCH_4_ for four regions. **a** The relative contributions of local CH_4_ emissions (local) and external CH_4_ emissions (transported) to the seasonal cycle of XCH_4_ in the four regions of interest (ROIs). These contributions are estimated using the Greenhouse Gas Framework–Flux (GHGF–Flux; see “Methods”) CH_4_ model. The description of the model and related analysis is presented in “Methods”. **b** The monthly averaged XCH_4_ in four ROIs: Northeast China, Southeast China, North Bangladesh, and North India, the same with the ROIs in Fig. 3a–d of Zhang et al.^1^. The error bars are the monthly uncertainties calculated by error propagation from the uncertainties in XCH_4_ retrievals in a certain month; zonal means which are the averaged XCH_4_ over the latitudinal band centered in the ROIs and the uncertainty estimate by one standard deviation in the shaded background are overlaid. The zonal latitudinal bands are 40°–50°N, 23°–33°N, 20°–30°N, and 20°–30°N, respectively, for the four ROIs. The correlation coefficients between the zonal means and regional means are 0.53, 0.50, 0.73, and 0.83, respectively, with all *p* values less than 0.01 from the significance tests of the linear regression relationship.

Zhang et al.^1^ also claimed that the spatial distribution of rice paddies was a major factor in determining the spatial XCH_4_ distributions in monsoon Asia based on their spatial consistencies. However, there is also spatial consistency between XCH_4_ and the non-agriculture fluxes, and cross-correlation between agriculture and non-agriculture CH_4_ fluxes. From our analysis based on the emission database for global atmospheric research (EDGAR) reanalysis data as shown in Fig. 2a, the spatial correlation between non-agriculture CH_4_ emissions and XCH_4_ in Monsoon Asia is higher than that between agriculture and XCH_4_. It indicates the rice paddy emission may not be the most important factor regulating the spatial distribution of XCH_4_ in Monsoon Asia. Moreover, the non-agriculture and agriculture CH_4_ emissions are strongly cross-correlated in space (Fig. 2b) since agricultural lands in Monsoon Asia are usually close to the non-agriculture sources, which mainly include anthropogenic sources (energy and fossil fuel production) and waste and wastewater sources^8^. Such a strong cross-correlation should be accounted for when inferring a relationship between the spatial structure of XCH_4_ and agricultural flux, given that the non-agriculture sources in Asia are also highly variable in both space and time^9^.

**Fig. 2:**
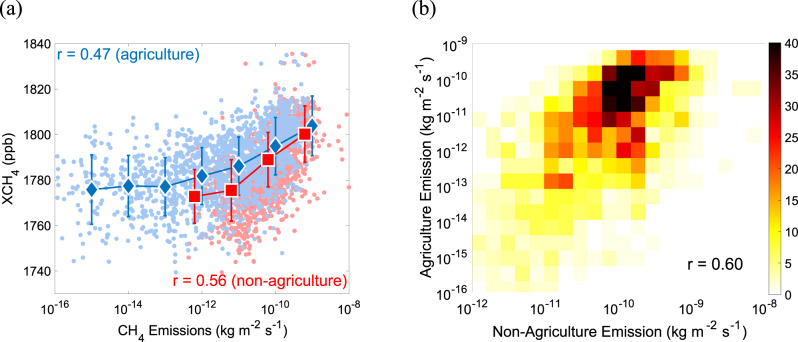
Correlations between emissions and column abundances. **a** The correlation between XCH_4_ observed by the space-based instrument SCanning Imaging Absorption spectroMeter for Atmospheric CHartographY (SCIAMACHY) in Monsoon Asia in 2010 (Supplementary Fig. 3) and the CH_4_ emissions from all sectors (Supplementary Fig. 4a) except agriculture (non-agriculture) and agriculture soil (agriculture; Supplementary Fig. 4b), respectively. The correlation coefficients (*r*) are also indicated. The error bar is defined as the standard deviation for each group of observations divided by the CH_4_ emissions. **b** The bivariable histogram between agriculture and non-agriculture CH_4_ emissions in Monsoon Asia in 2010. The correlation coefficient (0.60) is also indicated. The CH_4_ emission data are obtained from the Emission Database for Global Atmospheric Research (EDGAR) bottom-up inventory in 2010.

Altogether, our re-analysis of the XCH_4_ observations combined with atmospheric transport model simulations suggests the need for caution in using correlation-based inference to quantify the change of paddy rice CH_4_ emissions from the simple relationship between the area and growth of paddy rice and satellite-observed XCH_4_. We suggest that combining satellite observations and model simulations in a data assimilation system (e.g., ref. ^10^) is needed to disentangle the influence of local rice paddy emissions from other sources within the region and large scale advected signals.

## Methods

### Datasets

The IMAP v7.2 XCH_4_ data product from SCIAMACHY retrievals, which were downloaded from the ESA GHG-CCI data portal (http://www.esa-ghg-cci.org/). The XCH_4_ data from 2003 to 2011 are used for analysis in this study. The EDGAR methane emission bottom-up inventory data are obtained from The Emissions Database for Global Atmospheric Research (EDGAR) (https://edgar.jrc.ec.europa.eu/overview.php?v=432_GHG). The annual sector-specific grid map in 2010 for total CH4 flux and for agriculture soil is used in this study.

### Tagged Tracer simulations using GHGF-Flux simulation of CH4

Tagged tracer simulations were performed with the GHGF-Flux forward model. GHGF-Flux is a flux inversion system developed under NASA’s Carbon Monitoring System project. The GHGF is capable of simulating CH_4_, CO, CO_2_, and OCS and inherits the chemistry transport model from the GEOS-Chem. Chemical transport is driven by the Modern-Era Retrospective Analysis for Research and Applications, Version 2 (MERRA-2) meteorology produced with version 5.12.4 of the GEOS atmospheric data assimilation system^11^. To perform tracer transport, these fields are regridded to 2° × 2.5° horizontal resolution and archived with a temporal resolution of 3 h except for surface quantities and mixing depths, which have a temporal resolution of 1 h. Tracer transport is performed at 15 min time steps. Surface CH_4_ emissions were taken to be the total posterior CH_4_ flux from CarbonTracker-CH_4_ for 2010^10,12^, regridded to the 2° × 2.5° model resolution. Global OH fields were obtained from the Global Modeling Initiative model simulation run with MERRA reanalysis. With these sources and sinks XCH_4_ is simulated over 2010–2015 (using repeated 2010 surface fluxes). Simulations are performed with surface fluxes at every model grid cell and local fluxes only for the four ROIs, from which the local and transported XCH_4_ signals are isolated. For the XCH_4_ simulation the ROIs are approximated as latitude-longitude boxes (see Supplementary Fig. 1). The North China box is bounded by 45°–59°N and 128.75°–136.25°E; North India is bounded by 25°–35°N, 68.75°–81.25°E; the North Bangledesh is bounded by 19°–25°N and 86.25°–93.75°E; and Southeast China is bounded by 25°–29°N and 113.75°–121.75°E. The 6-year XCH_4_ time series are then detrended and averaged across the 6 years to obtain a mean seasonal cycle (see Supplementary Fig. 5). Note that simulated XCH_4_ is calculated using a column averaging kernel with a value of 1 for every level.

## Supplementary information

Supplementary Information

## Data Availability

CarbonTracker-CH_4_ results are provided by NOAA ESRL, Boulder, Colorado, USA (http://www.esrl.noaa.gov/gmd/ccgg/carbontracker-ch4/); The IMAP v7.2 data product from SCIAMACHY from the ESA-CCI data portal (http://www.esa-ghg-cci.org/); the EDGAR methane emission bottom-up inventory data are provided by the European Commission (https://edgar.jrc.ec.europa.eu/overview.php?v=432_GHG). Results from model simulations are available via an open-access link at 10.5281/zenodo.4291324.
