# Supplementary material for: Correlation between paddy rice growth and satellite-observed methane column abundance does not imply causation
Source: Nat Commun. 2021 Feb 19;12:1163. doi: 10.1038/s41467-021-21434-7 (PMC7895942; doi:10.1038/s41467-021-21434-7)
Supplement: Supplementary file 1 — Supplementary Information [file 41467_2021_21434_MOESM1_ESM.pdf]

Supplementary Information

Correlation between paddy rice growth and satellite-observed methane column abundance does not imply causation

Zhao-Cheng Zeng[1,2*], Brendan Byrne[3*], Fang-Ying Gong[2], Zhonghua He[4], and Liping Lei[5]

1 Joint Institute for Regional Earth System Science & Engineering, University of California, Los Angeles, Los Angeles, USA;
2 Division of Geological and Planetary Sciences, California Institute of Technology, Pasadena, USA;
3 Jet Propulsion Laboratory, California Institute of Technology, CA, USA;
4 Zhejiang Climate Center, Hanzhou, China;
5 Key Laboratory of Digital Earth Science, Institute of Remote Sensing and Digital Earth, Chinese Academy of Sciences, Beijing, China.

Corresponding to:
Z.-C. Zeng (zcz@gps.caltech.edu) and B. Byrne (brendan.k.byrne@jpl.nasa.gov)

Supplementary Figure 1.

Supplementary Figure 2.

Supplementary Figure 3.

Supplementary Figure 4.

Supplementary Figure 5.

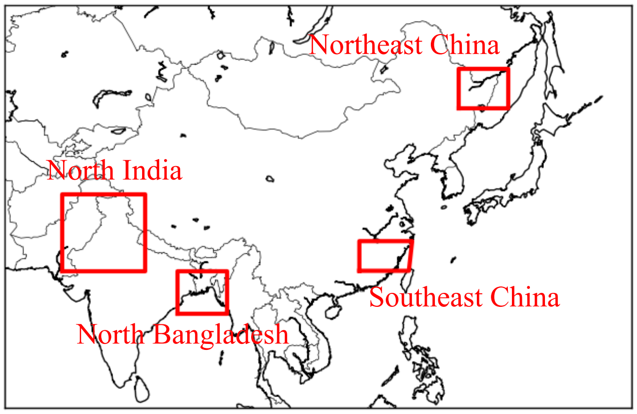

Supplementary Figure 1. The bounding boxes for the ROIs in model simulations. The Northeast China box is bounded by 45°-59° N and 128.75°-136.25° E; The North India is bounded by 25°-35° N, 68.75°-81.25° E; The North Bangladesh is bounded by 19°-25° N and 86.25°-93.75° E; and the Southeast China is bounded by 25°-29° N and 113.75°-121.75° E.

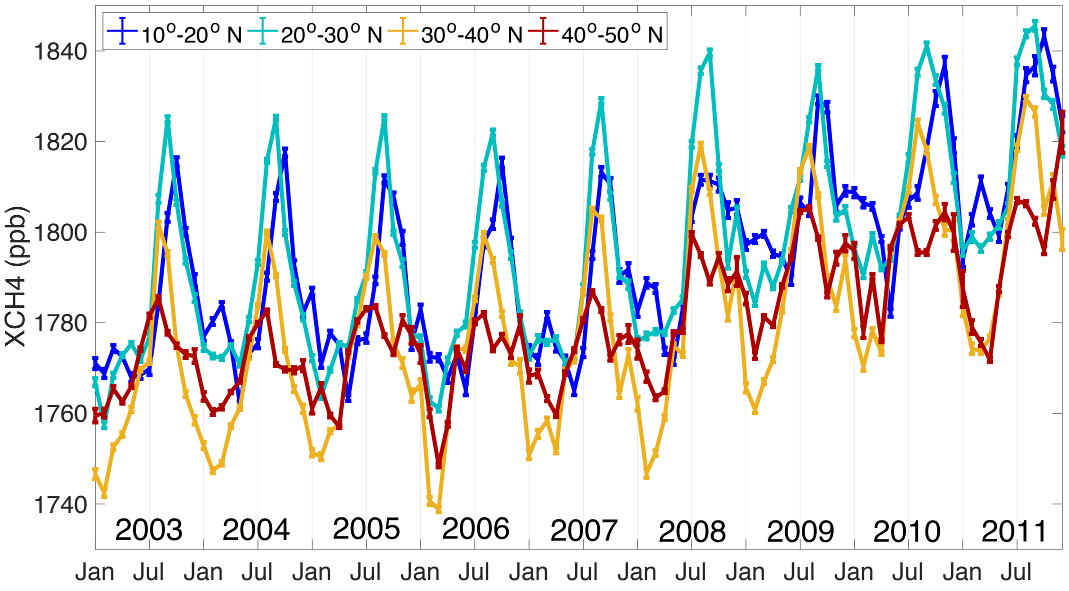

Supplementary Figure 2. The monthly mean XCH_4 from SCIAMACHY retrievals in Monsoon Asia for different latitude zones from 10° to 50° N. The error bars are the monthly uncertainties calculated by error propagation from the uncertainties in XCH_4 retrievals in a certain month.

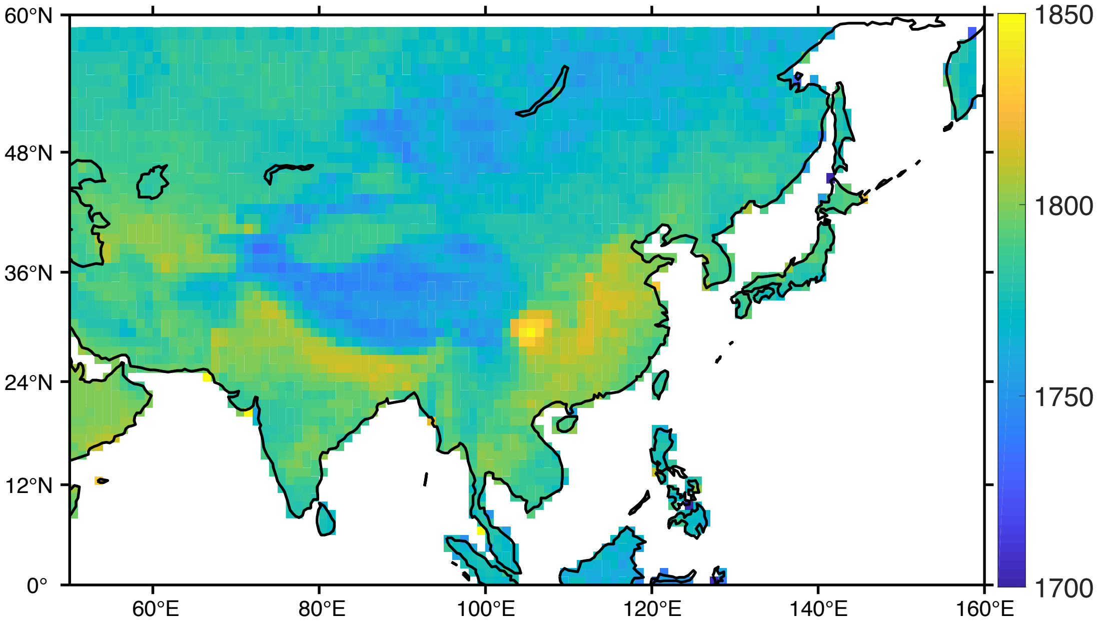

Supplementary Figure 3. Averaged XCH_4 (in unit of ppb) from SCIAMACHY retrievals in Monsoon Asia in 2010. The original retrievals were averaged into 1° × 1° grids in latitude and longitude.

(a)

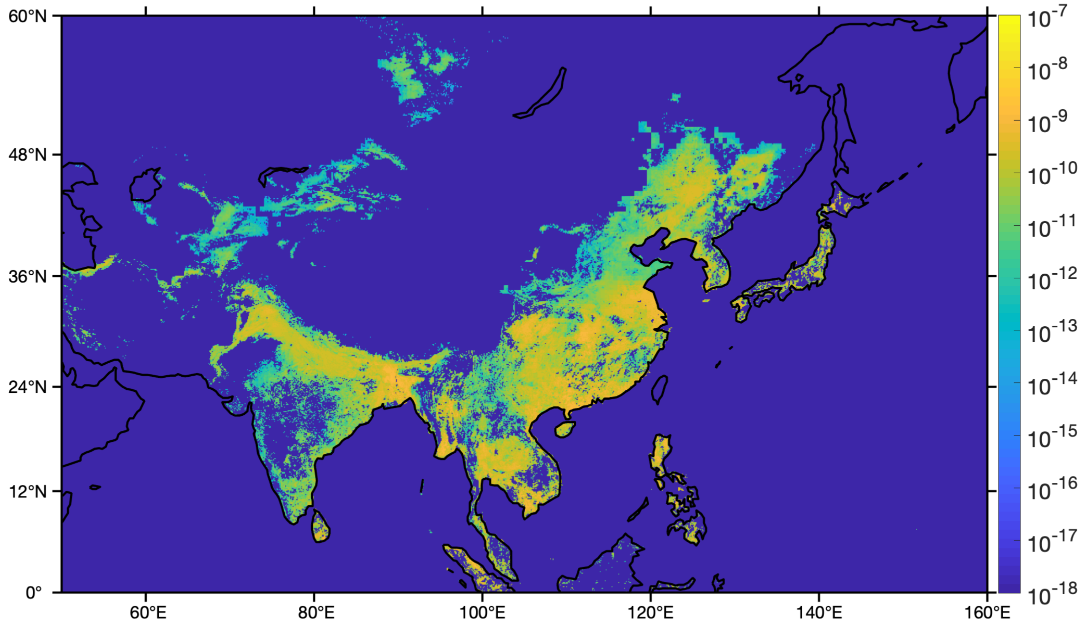

(b)

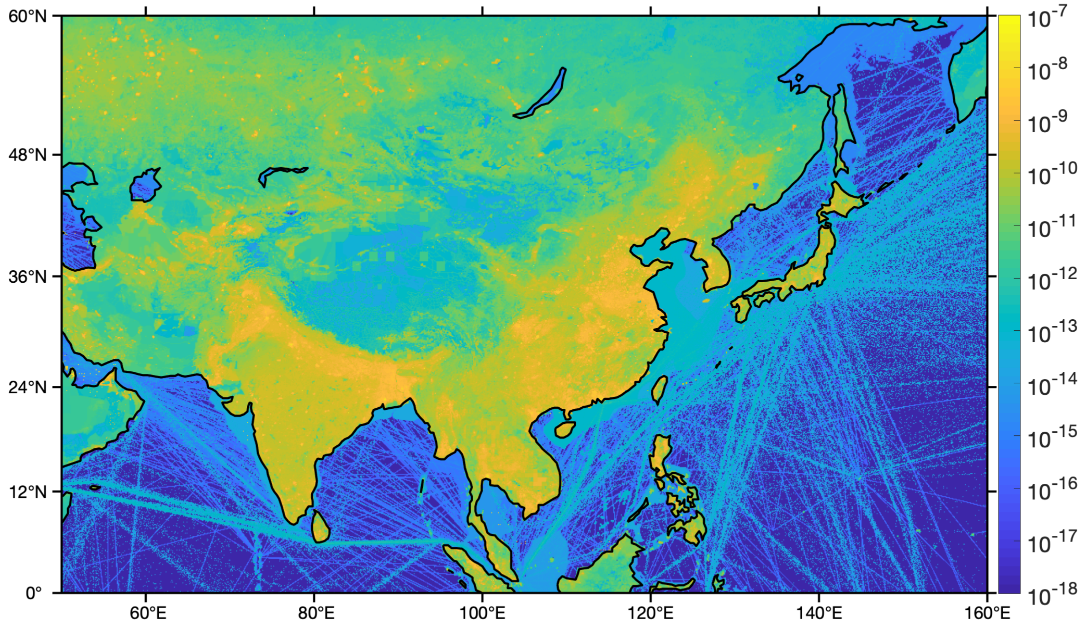

Supplementary Figure 4. CH_4 emissions (in unit of kg m^{-2} s^{-1}) from EDGAR bottom-up inventory for agriculture soil flux in (a) and total flux in (b) for the year of 2010.

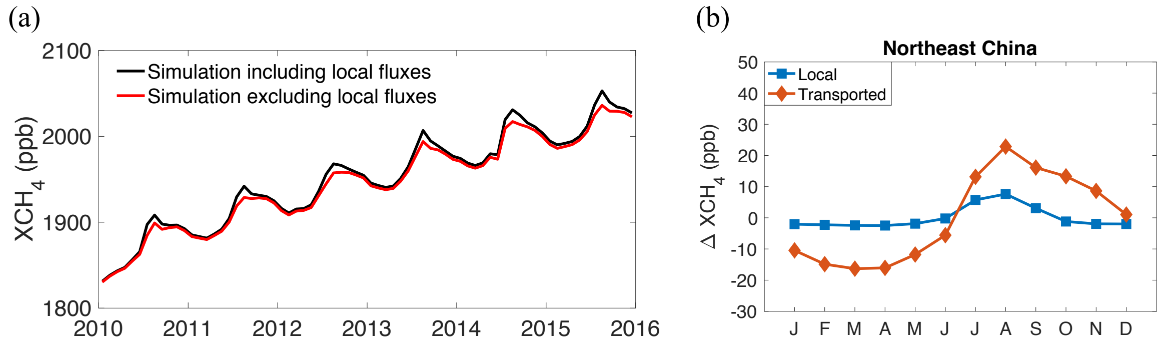

Supplementary Figure 5. GHGF-Flux simulated XCH_4 over Northeast China. (a) Monthly mean XCH_4 time series over six years for simulations including (black) and excluding (red) local fluxes; (b) The detrended six-year monthly mean time series of local (blue) and transported (red) contributions to the seasonal cycle of XCH_4, where the transported contribution is obtained from the simulation excluding local fluxes and the local contribution is the difference between detrended simulations with and without local fluxes.
